# Effects of ankle-foot prosthesis design on gait and standing performance in transfemoral prosthesis users: A scoping review

**DOI:** 10.1017/wtc.2025.10037

**Published:** 2026-01-21

**Authors:** Miguel A. Vaca, Molly Beestrum, Steven A. Gard, Matthew J. Major

**Affiliations:** 1 Department of Biomedical Engineering, Northwestern University, USA; 2 Jesse Brown Department of Veterans Affairs Medical Center, USA; 3 Department of Physical Medicine and Rehabilitation, Northwestern University Feinberg School of Medicine, USA; 4 Neurology Department, University of Minnesota, USA; 5 Galter Health Sciences Library, Northwestern University Feinberg School of Medicine, USA

**Keywords:** biomechanics, prosthetics, design

## Abstract

Ankle-foot mechanisms are designed to substitute for missing anatomical behavior of lower-limb prosthesis users. Historically, the majority of ankle-foot mechanism research has been focused on transtibial prosthesis users despite evidence that current knowledge is not directly translated to transfemoral prosthesis users, such as the influence of single-axis knee alignment during gait and the differences in standing balance management. This review attempts to characterize the current state of published knowledge about the effects of ankle-foot prosthesis design on standing and walking performance in transfemoral prosthesis users. The databases of PubMed, Embase, Cochrane Library, CINAHL, and IEEE Xplore were searched on January 6, 2025. Data from the selected articles were extracted and reported following the PRISMA extension for scoping reviews. Thirty-five articles were included that reported on seven different types of feet, ranging from simple designs like a solid ankle-cushioned heel (SACH) foot to more complex ones such as a microprocessor foot. The range of reported study tasks extended from standing and level walking to more complex tasks like incline/decline slopes and parcourse walking. The results suggest some parallels between transfemoral and transtibial prosthesis users, such as improvements with the incorporation of roll-over-shape (ROS) features and adaptation of a hydraulic ankle. The literature also emphasized how ankle-foot components affect ground force vector position and direction, influencing prosthetic knee control, highlighting the importance of considering the interaction between the prosthetic ankle-foot and knee mechanisms. Understanding these interactions will support the development of clinical practice guidelines by identifying the pair of prosthetic components that maximizes performance.

## 1. Introduction

The anatomical ankle-foot complex is a structure capable of adapting its mechanical behavior through neuromuscular control to meet the demands of a given task, such as maintaining stability while standing quietly or enabling efficient progression of the body over the stance limb while walking. During quiet standing, the muscles and ligaments of the foot and ankle work together to increase ankle-foot impedance to maintain upright posture (A. H. Hansen and Wang, 2010). During gait, the same muscles and ligaments alter foot and ankle stiffness and effective roll-over geometry to meet specific demands of the gait phases and adapt to different terrains (Andrew Howard Hansen, 2002; A. H. Hansen and Childress, 2005; A. H. Hansen et al., 2004). This behavior is reflected in the sagittal-plane ankle motion as the ankle joint angle alternates from plantarflexion to dorsiflexion and produces four distinct arcs of motion (Perry and Burnfield, 2010), the first three arcs corresponding to the stance phase (transitioning from plantarflexion to dorsiflexion back to plantarflexion) and the fourth arc (dorsiflexion) to the swing phase. During the stance phase, the ankle-foot functions include shock absorption, supporting body weight, enabling forward progression (Pace et al., 2020), and initiating the swing phase. During the swing phase, the ankle joint dorsiflexes to achieve safe limb progression and prepares for limb touchdown (Kent et al., 2021).

Lower-limb prosthesis users are clinically prescribed and fitted with prosthetic ankle-foot mechanisms that are designed to substitute for missing anatomical behavior. To achieve this aim, ankle-foot prosthesis designs have progressively become more complex, with vast amounts of studies comparing the advantages and disadvantages of each design iteration (van der Linde et al., 2004; Highsmith et al., 2016; Lathouwers et al., 2023). One example of a simple design is the solid ankle-cushioned heel (SACH) foot, which has a rigid ankle and foot structure that accommodates less-skilled ambulators (Macfarlane et al., 1997; Paradisi et al., 2015). Beyond the SACH foot, more complex devices have been developed (Matthew *J. Major and Stevens, 2024*) such as single-axis feet with a movable ankle joint that permits sagittal plane motion; multiaxial feet that permit motion in all three planes; dynamic response (DR) feet that are better able to store and return energy through the stance phase and pre-swing, respectively; and foot designs with a hydraulic damper at the ankle joint to modulate ankle impedance and accommodate to different terrains. Moreover, mechatronic components have been incorporated into ankle-foot prostheses to control their mechanical behavior and, in some cases, actuate ankle motion. Different ankle-foot prosthesis designs can have unique characteristics that ultimately affect the standing and walking performance of lower-limb prosthesis users (Wirta et al., 1991). Consequently, research has focused on characterizing stance-phase (i.e., loaded) mechanical characteristics of passive ankle-foot prostheses such as range of motion, damping, stiffness (M. J. Major et al., 2014; Vaca et al., 2022), and roll-over-shape (ROS) (Vaca et al., 2022) to map prosthesis mechanical properties to user outcomes (M. J. Major and Fey, 2017).

Transfemoral prosthesis users (TFPUs) and transtibial prosthesis users (TTPUs) are two primary cohorts that utilize prosthetic ankle-foot mechanisms for mobility. Although these devices serve essentially the same purpose for both groups (i.e., stance phase limb stability and progression), their mechanical function must be able to accommodate and support the needs associated with missing anatomical structures and mobility requirements unique to each cohort. Specific to TFPUs, ankle-foot mechanisms must also successfully interact with a prosthetic knee joint that, in most conventional prostheses, lacks volitional control (Matthew *J. Major and Stevens, 2024*). For instance, the geometry and mechanical response of the prosthetic ankle-foot while loaded directs the ground reaction force (GRF) vector position (Pace et al., 2020) that generates a desired extensor moment to prevent the mechanical knee from flexing (i.e., buckling) until an appropriate time during terminal stance (de Vries, 1995). Therefore, while studies have focused on the effects of the mechanical properties of ankle-foot prostheses on mobility outcomes in TTPUs (M. J. Major and Fey, 2017), those relationships may not directly translate to TFPUs due to their interaction with the prosthetic knee. For example, Barnett et al.(Barnett et al., 2021) reported an improvement in gait performance (more distance covered during a 2-minute walking test) while using a hydraulic ankle in conjunction with two different prosthetic knee designs, with even better mobility when that ankle-foot design is used in combination with a microprocessor knee. Additionally, Pace et al. (Pace et al., 2020) simulated the interaction effect of a single-axis knee alignment (center of rotation) and prosthetic ankle-foot stiffness on prosthetic limb stability during stance by analyzing critical prosthetic knee joint moments in the sagittal plane. Furthermore, evidence suggests that TFPUs manage their standing balance differently than TTPUs (Rougier and Bergeau, 2009; Toumi et al., 2021), which would similarly be affected by ankle-foot prosthesis mechanics (Nederhand et al., 2012).

The purpose of this review was to characterize the current state of published knowledge about the effects of ankle-foot prosthesis design on standing and walking performance in TFPUs. Results from this work will help establish our current understanding of ankle-foot prosthesis effects on clinically relevant outcomes in TFPUs and increase our knowledge of these relationships for leg prosthesis users by complementing the existing knowledge for TTPUs. Through this study, we also aim to identify gaps in this knowledge to direct research efforts that will ultimately inform clinical practice guidelines for people with lower-limb loss to improve outcomes for users of ankle-foot prostheses.

## 2. Methods

The protocol was developed by a three-member research team in collaboration with a research librarian and utilized the Preferred Reporting Items for Systematic reviews and Meta-Analyses extension for Scoping Reviews (PRISMA-ScR) as a guide to standardize reporting of the methods and results (Tricco et al., 2018).

### 2.1. Search strategy

The search strategy was developed according to the population–intervention–outcome (PIO) structure of global key terms: population – TFPUs; intervention – ankle-foot prosthesis; outcome – gait and standing balance. These PIO key terms were translated into search terms, including synonyms, alterations in spelling, and wildcards, and joined together using Boolean operators. The databases of PubMed, MEDLINE, Embase, Cochrane Library, Cumulative Index to Nursing and Allied Health Literature (CINAHL), and Institute of Electrical and Electronics Engineers (IEEE) Xplore were searched on January 6, 2025, and selected to retrieve articles from various disciplines, including health and engineering research. The search string was first developed for PubMed and then modified for use in other databases (Table 1). There was no date or language limit assigned on the search engine. A secondary search was performed by reviewing the references of those articles that were ultimately included in the review and relevant literature reviews.

**Table 1.** Search strings for the different databases

PubMed	
(“above knee” OR “above-knee” OR “above the knee” OR Transfemoral OR Trans-femoral OR “knee disarticulation”) AND (prosthes* OR prosthet* OR “artificial limbs”[MeSH] OR artificial-limb*) AND (foot OR ankle-foot OR feet OR ankle) AND (gait OR gait[MeSH] OR mobility OR walking[MeSH] OR walk* OR “Standing Position”[Mesh] OR “Postural Balance”[Mesh] OR “standing balance” OR “postural control” OR “quiet standing” OR “balance” OR “stability” OR “ambulation”)	
Embase	
(“above knee” OR “above-knee” OR “above the knee” OR transfemoral OR “trans femoral” OR “knee disarticulation”/exp OR “knee disarticulation”) AND (“limb prosthesis”/exp OR “limb prosthesis” OR prosthes* OR prosthet* OR “artificial limbs”/exp OR “artificial limbs”) AND (“foot”/exp OR “foot” OR “ankle”/exp OR “ankle” OR “foot ankle” OR feet OR ankle*) AND (“walking”/exp OR “walking” OR “gait”/exp OR “gait” OR “body equilibrium”/exp OR “body equilibrium” OR “standing”/exp OR “standing” OR gait* OR “mobility”/exp OR mobility OR walk* OR “standing balance”/exp OR “standing balance” OR “postural control”/exp OR “postural control” OR “quiet standing” OR “balance”/exp OR “balance” OR “stability”/exp OR “stability” OR “ambulation”/exp OR “ambulation”)	
Cochrane	
((“above knee” OR “above-knee” OR “above the knee” OR Transfemoral OR Trans-femoral OR “knee disarticulation”)):ti,ab,kw	[S; Limits]
MeSH descriptor: [Artificial Limbs] explode all trees	[MeSH]
((prosthes* OR prosthet* OR artificial-limb*)):ti,ab,kw	[S; Limits]
#2 OR #3	[Limits]
((foot OR ankle-foot OR feet OR ankle)):ti,ab,kw	[S; Limits]
MeSH descriptor: [Gait] explode all trees	[MeSH]
MeSH descriptor: [Walking] explode all trees	[MeSH]
MeSH descriptor: [Postural Balance] explode all trees	[MeSH]
MeSH descriptor: [Standing Position] explode all trees	[MeSH]
((gait OR mobility OR walk* OR “standing balance” OR “postural control” OR “quiet standing” OR “balance” OR “stability” OR “ambulation”)):ti,ab,kw	[S; Limits]
#6 OR #7 OR #8 OR #9 OR #10	[Limits]
#1 AND #4 AND #5 AND #11	[Limits]
CINAHL	
(“above knee” OR “above-knee” OR “above the knee” OR Transfemoral OR Trans-femoral OR “knee disarticulation”) AND (MH “Limb Prosthesis” OR prosthes* OR prosthet* OR artificial-limb*) AND (foot OR ankle-foot OR feet OR ankle*) AND (MH “Walking” OR MH “Gait” OR MH “Balance, Postural” OR MH “Standing” OR gait* OR mobility OR walk* OR “standing balance” OR “postural control” OR “quiet standing” OR “balance” OR “stability” OR “ambulation”)	
IEEE Xplore	
“above knee” OR “above-knee” OR “above the knee” OR Transfemoral OR Trans-femoral OR “knee disarticulation”	[in Metadata]
[AND] prosthes* OR prosthet* OR artificial-limb*	[in Metadata]
[AND] foot OR ankle-foot OR feet OR ankle*	[in Metadata]
[AND] gait* OR mobility OR walk* OR “standing balance” OR “postural control” OR “quiet standing” OR “balance” OR “stability” OR “ambulation”	[in Metadata]

### 2.2. Selection criteria

The screening and selection processes were performed primarily by a single reviewer. The retrieved records from all databases were uploaded into a central database (EndNote v20.0.1, Philadelphia, PA, USA), and duplicates were removed. The title and abstract of the remaining records were first screened for relevance. If relevant, the full text of those articles was evaluated against the inclusion/exclusion criteria. In cases of uncertainty about the inclusion of a specific article, the primary reviewer and two senior authors reviewed and discussed the article’s contents to arrive at a final decision. The inclusion criteria included: original research studies involving human subject experiments or numerical simulations; studies examining the population of adult TFPUs (18 years or older); interventions comparing different prosthetic ankle-foot mechanisms (either defined by design type or quantified user-independent mechanical properties); outcomes quantifying a dimension of gait and/or balance. The exclusion criteria included: meta-analysis, systematic reviews, group consensus, and individual opinions; studies that compared only prosthetic knees without modifications to the ankle-foot prosthesis; and studies that present combined results for TFPUs and TTPUs that did not allow separate analyses of each group. The articles had to be written in English or translated to the English language for inclusion, and there were no restrictions on amputation etiology, participant experience or activity level, tested prosthetic knee, year of publication, or experimental setting (e.g., clinic, laboratory, in silico, etc.).

### 2.3. Data extraction

Data to characterize the body of literature and address the research aim were extracted from the included articles and compiled into an electronic spreadsheet (Excel v1206, Microsoft, Redmond, WA, USA). These data elements included authors, publication year, article title, article type, publication journal, study design, setting if human subject experiments, stated aims, protocol, prosthetic device interventions, prosthesis manufacturer(s) as appropriate, primary outcome measures, statistical analysis methods, participant characteristics as appropriate (sample size, amputation level(s), mean or range of age, sex distribution, amputation etiology distribution), and key results and findings.

## 3. Results

**Figure 1.**
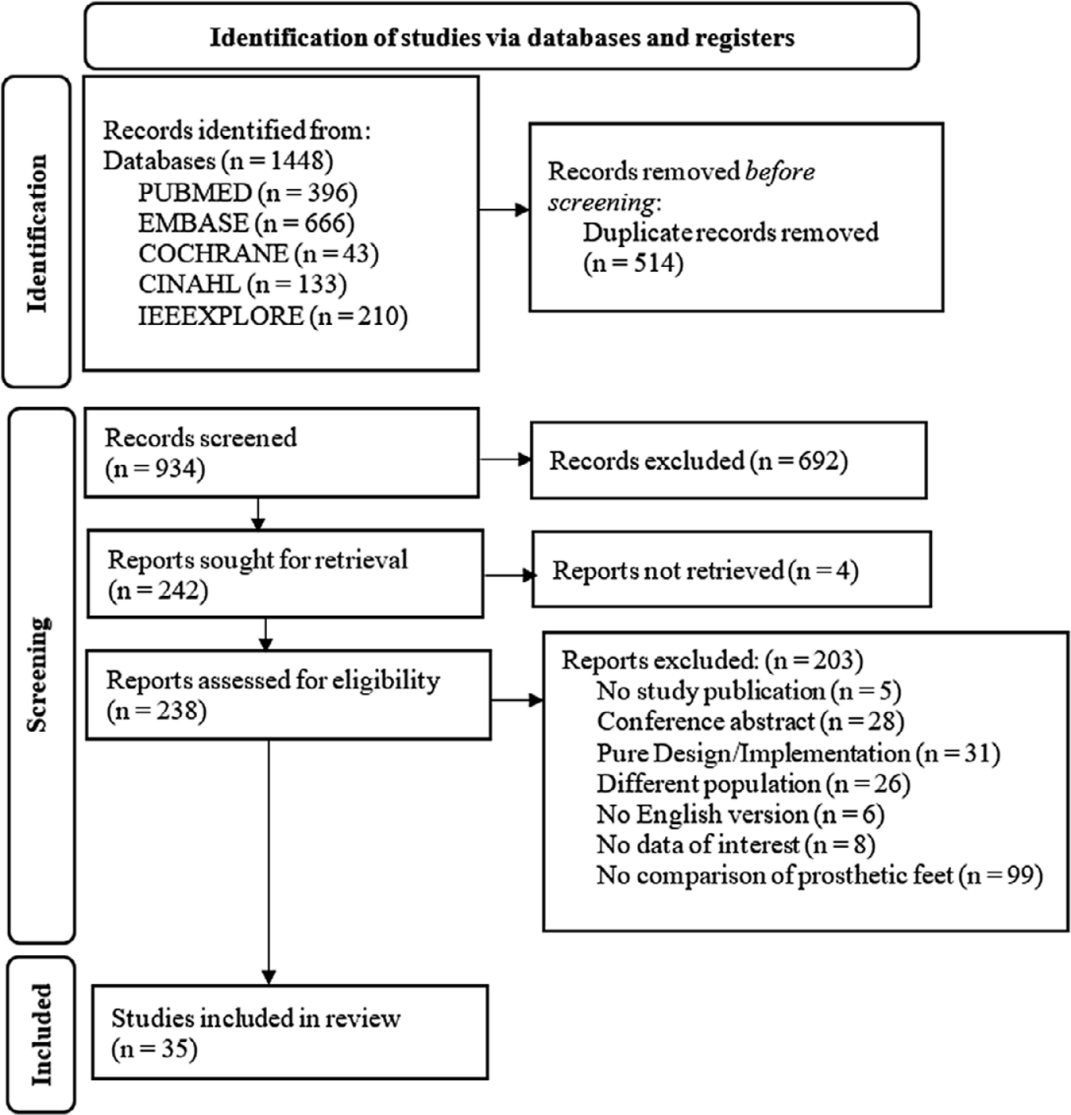
PRISMA flow diagram of screening and evaluation results.

**Figure 2.**
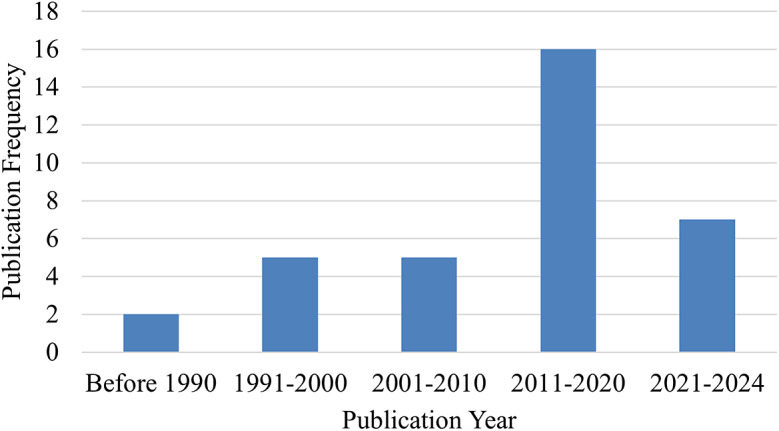
Publication frequency of year published.

**Figure 3.**
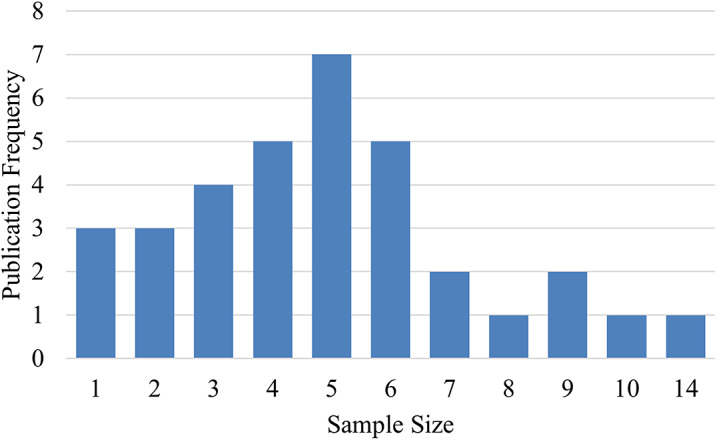
Publication frequency of sample size for in vivo studies.

**Figure 4.**
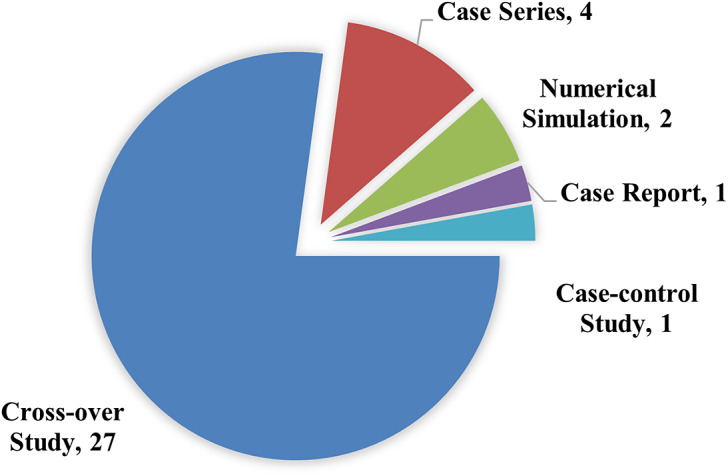
Proportion of study designs across reviewed literature.

The final search yielded a total of 1448 articles prior to screening. Following screening and evaluation, 35 articles were ultimately included in the review (Figure 1). These 35 articles were published over 39 years (1984 to 2024), with more than half (n = 23) published in the latter 14 years (Figure 2). Two studies included in-silico analysis (Sensinger et al., 2013; Pace et al., 2020), and the number of participants for human subject studies ranged from 1 to 14 (Figure 3). The study designs employed were also diverse (Figure 4), with the vast majority (n = 27) utilizing a cross-over repeated-measures approach. A summary of the participant characteristics is presented in Table 2.

**Table 2.** Participant characteristics

Data	Range	Number of studies reporting
Sample size^a^ (Goh et al., 1984; James and Stein, 1986; Boonstra et al., 1993; Macfarlane et al., 1997; Blumentritt et al., 1998; van der Linden et al., 1999; Goujon et al., 2006; Graham et al., 2007; McNealy and Gard, 2008; Graham et al., 2008; Lee and Hong, 2009; Sedki and Moore, 2013; Sensinger et al., 2013; Ingraham et al., 2014; De Asha et al., 2014; Ingraham et al., 2016; Klenow et al., 2016; Ernst et al., 2017; Bai et al., 2017, 2018; Armannsdottir et al., 2018; Alexander et al., 2018; De Pauw et al., 2018; McGrath et al., 2018; Alexander et al., 2020; De Pauw et al., 2020; Barnett et al., 2021; Agrawal et al., 2022; Ernst et al., 2022; Hong et al., 2022; Lathouwers et al., 2022; Pröbsting et al., 2022; Elke Lathouwers et al., 2023)	1–14	35
Age (years) (Goh et al., 1984; James and Stein, 1986; Blumentritt et al., 1998; Boonstra et al., 1993; Macfarlane et al., 1997; van der Linden et al., 1999; Goujon et al., 2006; Graham et al., 2007; Graham et al., 2008; McNealy and Gard, 2008; Lee and Hong, 2009; Sedki and Moore, 2013; Sensinger et al., 2013; Ingraham et al., 2014; Ingraham et al., 2016; Klenow et al., 2016; Ernst et al., 2017; Bai et al., 2017, 2018; McGrath et al., 2018; Alexander et al., 2018; Armannsdottir et al., 2018; De Pauw et al., 2018; Alexander et al., 2020; De Pauw et al., 2020; Barnett et al., 2021; Agrawal et al., 2022; Ernst et al., 2022; Hong et al., 2022; Lathouwers et al., 2022; Pröbsting et al., 2022; Elke Lathouwers et al., 2023)	18–70	32
Sex distribution (% male) (James and Stein, 1986; Boonstra et al., 1993; Macfarlane et al., 1997; Macfarlane et al., 1997; van der Linden et al., 1999; Goujon et al., 2006; Graham et al., 2007; Graham et al., 2008; McNealy and Gard, 2008; Lee and Hong, 2009; Sedki and Moore, 2013; Sensinger et al., 2013; Ingraham et al., 2014, Ingraham et al., 2016; Klenow et al., 2016; Bai et al., 2017, 2018; Alexander et al., 2018; Armannsdottir et al., 2018; McGrath et al., 2018; De Pauw et al., 2018; Alexander et al., 2020; De Pauw et al., 2020; Barnett et al., 2021; Hong et al., 2022; Lathouwers et al., 2022; Pröbsting et al., 2022; Agrawal et al., 2022; Elke Lathouwers et al., 2023)	50–100	29
Etiology (% traumatic^b^) (Boonstra et al., 1993; Macfarlane et al., 1997; Macfarlane et al., 1997; Blumentritt et al., 1998; Goujon et al., 2006; Graham et al., 2007; Graham et al., 2008; McNealy and Gard, 2008; Lee and Hong, 2009; Sensinger et al., 2013; De Asha et al., 2014; Klenow et al., 2016; Alexander et al., 2018; Armannsdottir et al., 2018; Barnett et al., 2021; Ernst et al., 2022; Lathouwers et al., 2022; Elke Lathouwers et al., 2023)	50–100	18

a For those studies that included transtibial and transfemoral prosthesis users, this number represents only the transfemoral cohort.

b Other etiologies included vascular, sarcoma, infection, arterial occlusion.

For those articles that performed human subject testing with commercially available ankle-foot prostheses, these devices spanned a range of classifications: solid-ankle feet (n = 21) (Goh et al., 1984; Macfarlane et al., 1997; Macfarlane et al., 1997; Blumentritt et al., 1998; Goujon et al., 2006; McNealy and Gard, 2008; Lee and Hong, 2009; Sedki and Moore, 2013; Sensinger et al., 2013; De Asha et al., 2014; Klenow et al., 2016; Bai et al., 2017; Ernst et al., 2017; Alexander et al., 2018; Bai et al., 2018; De Pauw et al., 2018; McGrath et al., 2018; Alexander et al., 2020; De Pauw et al., 2020; Barnett et al., 2021; Pröbsting et al., 2022); single-axis feet (n = 13) (Goh et al., 1984; James and Stein, 1986; Blumentritt et al., 1998; Lee and Hong, 2009; Sensinger et al., 2013; De Asha et al., 2014; Klenow et al., 2016; Bai et al., 2017, 2018; Alexander et al., 2018; McGrath et al., 2018; Alexander et al., 2020; Barnett et al., 2021); DR feet (n = 23) (Boonstra et al., 1993; Blumentritt et al., 1998; Macfarlane et al., 1997; van der Linden et al., 1999; Goujon et al., 2006; Graham et al., 2007; Graham et al., 2008; McNealy and Gard, 2008; Sedki and Moore, 2013; De Asha et al., 2014; Klenow et al., 2016; Bai et al., 2017, 2018; Alexander et al., 2018; McGrath et al., 2018; Alexander et al., 2020; Barnett et al., 2021; Agrawal et al., 2022; Ernst et al., 2022; Lathouwers et al., 2022; Pröbsting et al., 2022; Elke Lathouwers et al., 2023); multiaxial feet (n = 6) (Boonstra et al., 1993; Blumentritt et al., 1998; van der Linden et al., 1999; Graham et al., 2007; Graham et al., 2008; McNealy and Gard, 2008); feet with a hydraulic ankle joint (n = 6) (Sedki and Moore, 2013; De Asha et al., 2014; Bai et al., 2017, 2018; McGrath et al., 2018; Barnett et al., 2021); microprocessor feet without power generation (n = 5) (Ernst et al., 2017; Bai et al., 2018; De Pauw et al., 2018; De Pauw et al., 2020; Ernst et al., 2022); and powered/actuated feet (n = 2) (Armannsdottir et al., 2018; Pröbsting et al., 2022). Additionally, nine articles (James and Stein, 1986; Lee and Hong, 2009; K. A. Ingraham et al., 2014; K. M. Ingraham et al., 2016; De Pauw et al., 2018; De Pauw et al., 2020; Hong et al., 2022; Lathouwers et al., 2022; Elke Lathouwers et al., 2023) reported comparisons between different foot behaviors using experimental devices that were not commercially available. These experimental devices were used to assess effects of foot curvature (Hong et al., 2022), “ankle” range of motion (Lee and Hong, 2009), keel motion (James and Stein, 1986), increase in ankle stiffness (K. A. Ingraham et al., 2014; K. M. Ingraham et al., 2016; De Pauw et al., 2018; De Pauw et al., 2020; Lathouwers et al., 2022; Elke Lathouwers et al., 2023), and powered plantarflexion (K. A. Ingraham et al., 2014; K. M. Ingraham et al., 2016). Across all studies (human subject and numerical simulation), 29 studies (Goh et al., 1984; James and Stein, 1986; Boonstra et al., 1993; Macfarlane et al., 1997; Blumentritt et al., 1998; van der Linden et al., 1999; McNealy and Gard, 2008; Lee and Hong, 2009; Goujon et al., 2006; Graham et al., 2007; Graham et al., 2008; Sedki and Moore, 2013; Sensinger et al., 2013De Asha et al., 2014; Klenow et al., 2016; Bai et al., 2017, 2018; De Pauw et al., 2018; Alexander et al., 2018; Armannsdottir et al., 2018; Alexander et al., 2020; De Pauw et al., 2020; Agrawal et al., 2022; Ernst et al., 2022; Hong et al., 2022; Pröbsting et al., 2022Lathouwers et al., 2022; Elke Lathouwers et al., 2023) observed effects of only changing ankle-foot prosthesis design/function, while six studies (K. A. Ingraham et al., 2014; K. M. Ingraham et al., 2016; Ernst et al., 2017; McGrath et al., 2018; Pace et al., 2020; Barnett et al., 2021) observed effects of changing properties of both the prosthetic ankle-foot mechanism and knee.

**Figure 5.**
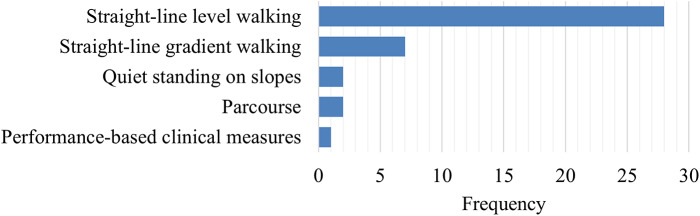
Frequency of study tasks across reviewed literature.

Performance in a range of tasks was assessed, classified into five categories (Figure 5):

1. Straight-line level walking (n = 29) (Goh et al., 1984; Boonstra et al., 1993; Macfarlane et al., 1997; Blumentritt et al., 1998; van der Linden et al., 1999; Goujon et al., 2006; Graham et al., 2007; Graham et al., 2008; McNealy and Gard, 2008; Lee and Hong, 2009; Sensinger et al., 2013; De Asha et al., 2014; Ingraham et al., 2014; K. M. Ingraham et al., 2016; Klenow et al., 2016; Bai et al., 2017; De Pauw et al., 2018; Alexander et al., 2018; Armannsdottir et al., 2018; Pace et al., 2020; Alexander et al., 2020; De Pauw et al., 2020; Barnett et al., 2021; Agrawal et al., 2022; Hong et al., 2022; K. A. Lathouwers et al., 2022; Pröbsting et al., 2022; Elke Lathouwers et al., 2023);
2. Straight-line gradient walking (n = 7), including sagittal plane (incline/decline) (James and Stein, 1986; Alexander et al., 2018; Bai et al., 2018; Alexander et al., 2020; Ernst et al., 2022; Lathouwers et al., 2022) and frontal plane (camber) (Bai et al., 2017);
3. Quiet standing on slopes (n = 2) (Ernst et al., 2017; McGrath et al., 2018);
4. Parcourse (circuit of different terrain conditions) walking (n = 2) (Graham et al., 2007; Barnett et al., 2021);
5. Performance-based clinical outcome measures (n = 1) (Barnett et al., 2021), including the two-minute walk test, timed-up-and-go, and L-test of functional mobility.

**Figure 6.**
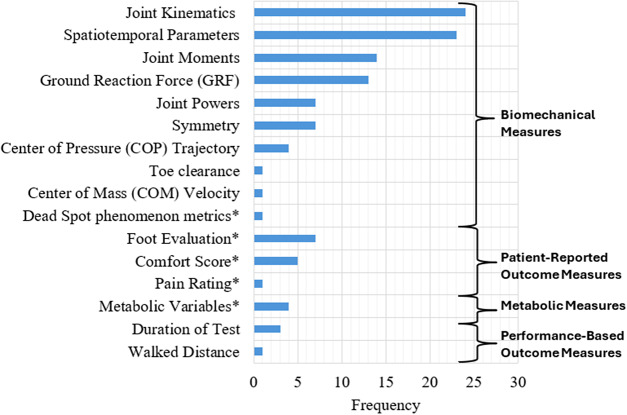
Frequency of outcome variable groups. *Dead spot phenomenon metrics include variables that quantify a disruption in forward progression of plantar center-of-pressure under the prosthetic ankle-foot systems; foot evaluation refers to any questionnaire that intend to evaluate the participant’s perception of each condition such as Seattle Prosthesis Evaluation Questionnaire (PEQ) and custom-designed questionnaires; comfort score refers to questionnaires that evaluate the user’s perceived comfort of the prosthesis; pain rating refers to questionnaires that record the subject’s pain rating; metabolic variables include any variable related to metabolic consumption, that is, oxygen consumption and heart rate.

**Figure 7.**
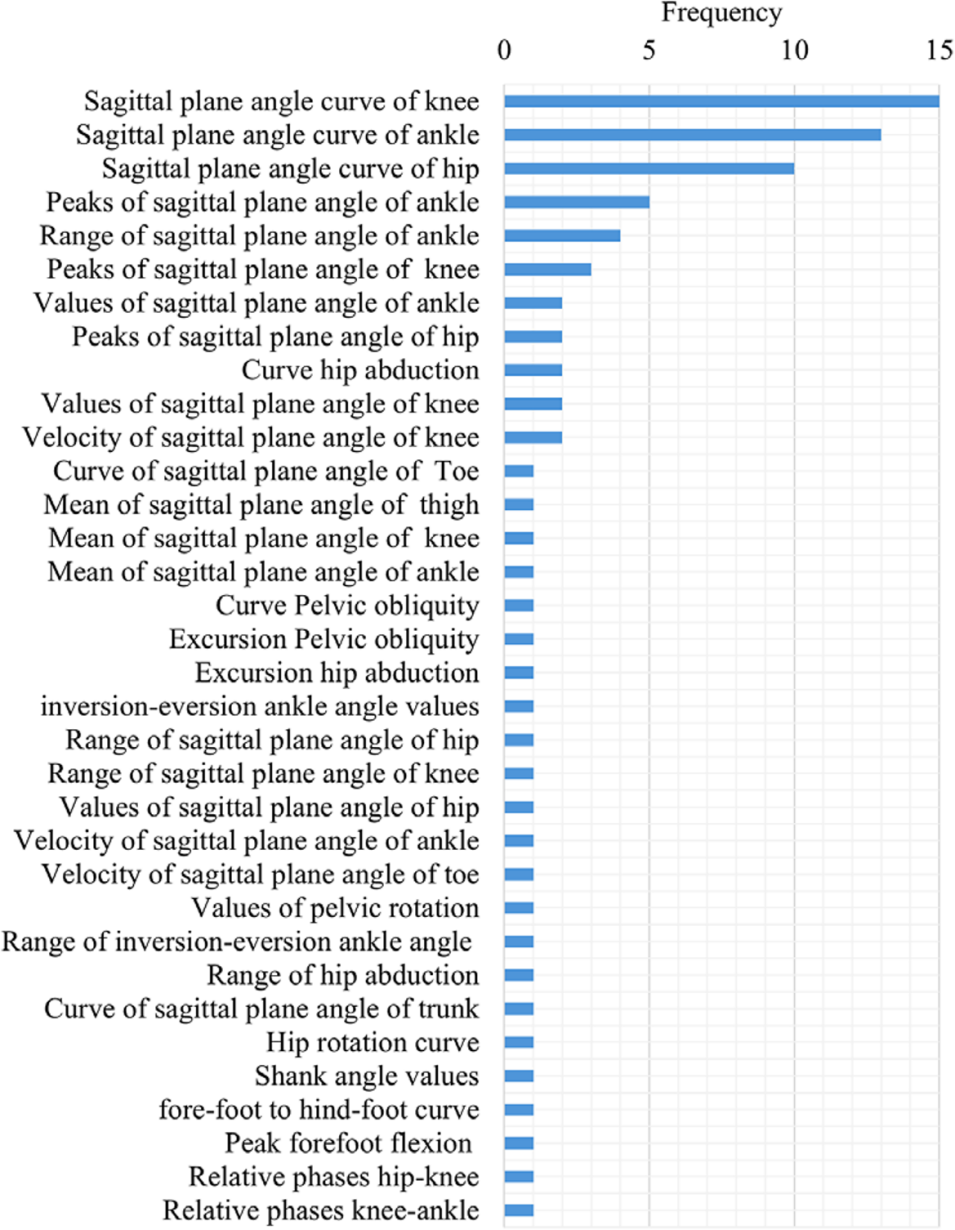
Frequency of individual outcome variables that are part of joint kinematics.

**Figure 8.**
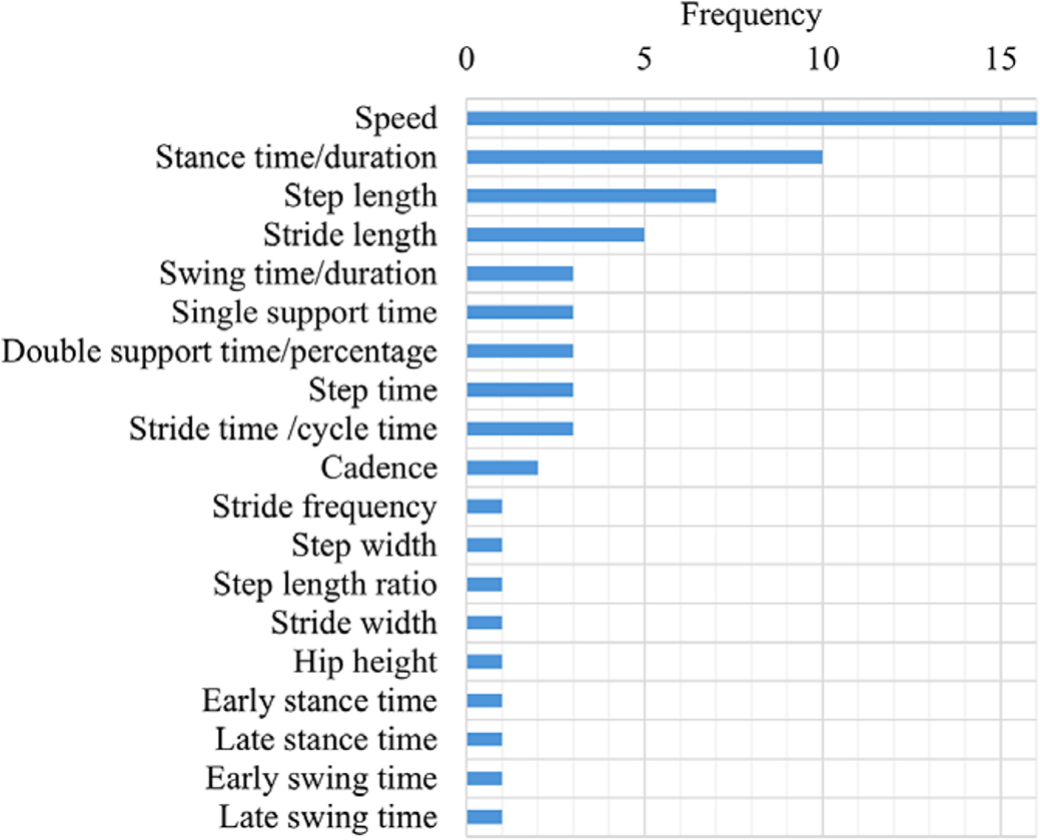
Frequency of individual outcome variables that are part of spatiotemporal parameters.

**Figure 9.**
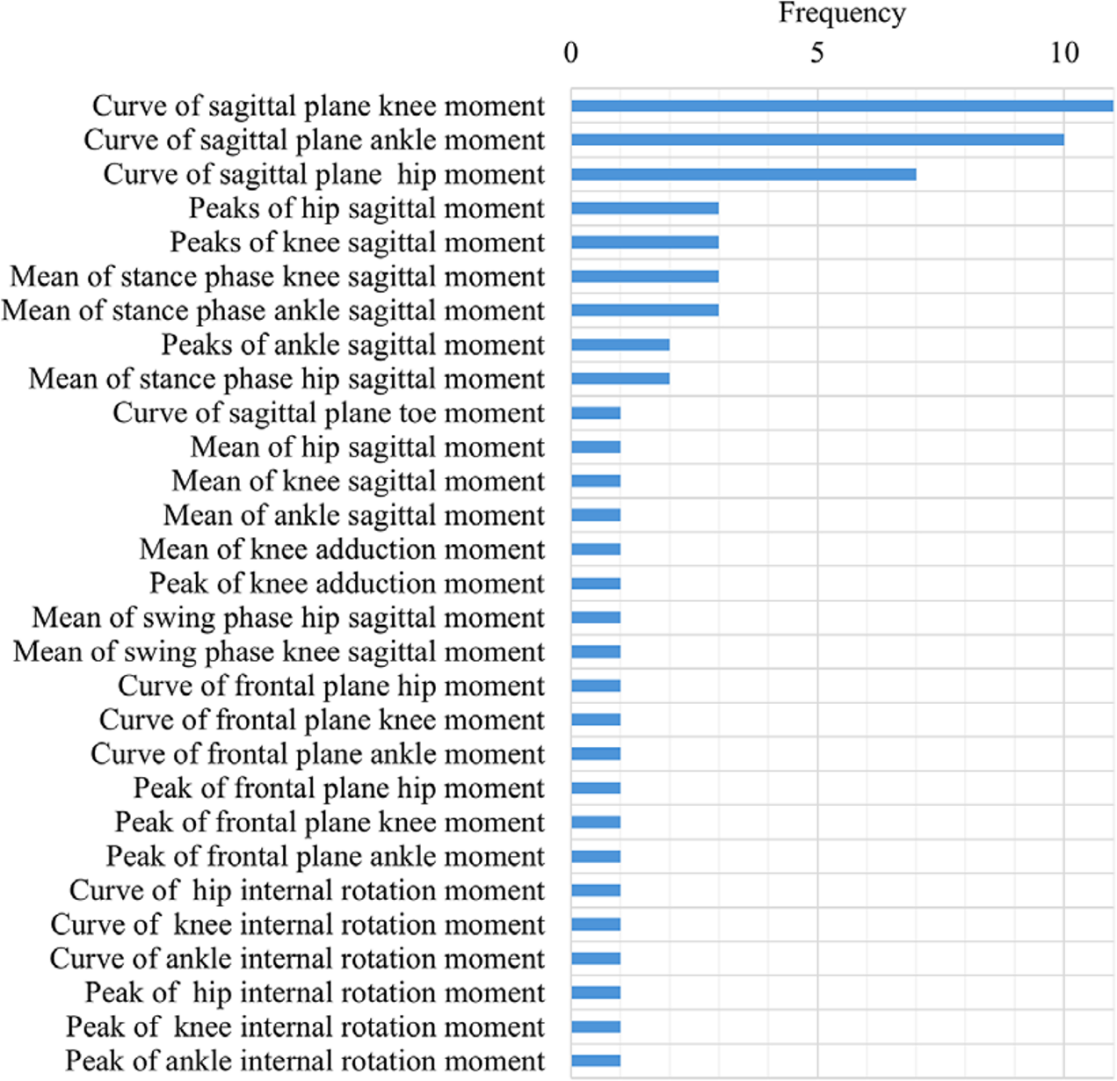
Frequency of individual outcome variables that are part of joint moments.

**Figure 10.**
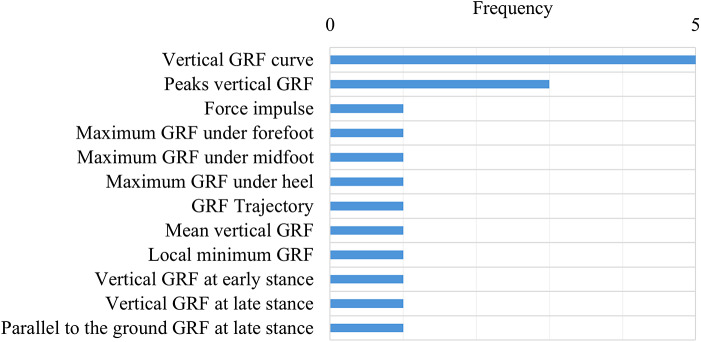
Frequency of individual outcome variables that are part of ground reaction forces.

While performance-based outcome measures and parcourse conditions could include elements of categories 1–3, these were categorized separately given their specific and unique protocols. The main characteristics of the unique protocols were extracted and classified by task and used medium (i.e., level ground, treadmill, ramp, etc.). These results were summarized and presented in Table 3. Across all the studies, the individual outcome variables were classified into 16 outcome variable groups and quantified, including biomechanical, spatiotemporal, and metabolic metrics (Figure 6), with nine articles (Boonstra et al., 1993; Blumentritt et al., 1998; Graham et al., 2007; McNealy and Gard, 2008; Sedki and Moore, 2013; Bai et al., 2017, 2018; Armannsdottir et al., 2018; De Pauw et al., 2018) that utilized patient-reported outcome measures to capture participant’s perception. A table with the list of individual outcome variables reported by each selected study and classified by outcome variable group is included as supplementary material (Supplemental Material 1: individual variables for each study). The frequency of individual outcome variables for the most frequently reported groups is included in Figures 7–10 (joint kinematics – Figure 7, spatiotemporal parameters – Figure 8, joint moments – Figure 9, and ground reaction forces – Figure 10). Key findings from each article are presented in Table 4.

**Table 3.** Methodology Characteristics

Tasks	Task medium	Methods	Range	Number of studies reporting
Straight-line level walking	Level ground	Walkway length (Boonstra et al., 1993; van der Linden et al., 1999; Goujon et al., 2006; De Asha et al., 2014; K. A. Ingraham et al., 2014; K. M. Ingraham et al., 2016; Klenow et al., 2016; Bai et al., 2017; Agrawal et al., 2022)	7–10 m	9
		Course length (Goh et al., 1984; Macfarlane et al., 1997; Macfarlane et al., 1997)	20–50 m	3
		Instrumented ramp length (Alexander et al., 2018; Alexander et al., 2020)	6 m	2
		Self-selected comfortable speed (Goh et al., 1984; Boonstra et al., 1993; van der Linden et al., 1999; Goujon et al., 2006; Graham et al., 2007; McNealy and Gard, 2008; De Asha et al., 2014; K. A. Ingraham et al., 2014; K. M. Ingraham et al., 2016; Bai et al., 2017; Alexander et al., 2018; Armannsdottir et al., 2018; Alexander et al., 2020; Pröbsting et al., 2022)	0.55–1.55 m/s	14
		Self-selected fast speed (Boonstra et al., 1993; McNealy and Gard, 2008; Bai et al., 2018; Pröbsting et al., 2022)	0.80–1.69 m/s	4
		Assigned speed (Macfarlane et al., 1997; Macfarlane et al., 1997; Lee and Hong, 2009)	0.67–1.56 m/s	3
		Fastest speed you can safely walk (Graham et al., 2007)	1.40–1.69 m/s	1
		Close to fastest speed (van der Linden et al., 1999)	1.45–1.39 m/s	1
		Self-selected slow speed (McNealy and Gard, 2008)	0.21–0.66 m/s	1
	Treadmill	Self-selected comfortable speed treadmill (Boonstra et al., 1993; De Pauw et al., 2018; Lathouwers et al., 2022; Elke Lathouwers et al., 2023)	0.41–1.35 m/s	4
		Self-selected fast speed treadmill (Boonstra et al., 1993)	1.05–1.69 m/s	1
		Fast: 125% self-selected comfortable speed (De Pauw et al., 2018; Lathouwers et al., 2022; Elke Lathouwers et al., 2023)	0.53–1.19 m/s	3
		Slow:75% self-selected comfortable speed (De Pauw et al., 2018; Lathouwers et al., 2022; Elke Lathouwers et al., 2023)	0.30–1.16 m/s	3
Straight-line gradient walking	Ramp	Length (Alexander et al., 2018; Bai et al., 2018; Alexander et al., 2020; Ernst et al., 2022)	3 – 6 m	4
		Inclination (Alexander et al., 2018; Bai et al., 2018; Alexander et al., 2020; Ernst et al., 2022)	15° downhill to 12°uphill	4
		Self-selected speed downhill (Alexander et al., 2018; Bai et al., 2018; Alexander et al., 2020)	0.81–1.57 m/s	3
		Self-selected speed uphill (Alexander et al., 2018; Bai et al., 2018)	0.58–1.45 m/s	2
	Treadmill	Inclination (James and Stein, 1986; Lathouwers et al., 2022)	10.00% or 19° downhill to 16°uphill	2
		Assigned speed (James and Stein, 1986)	0.48–0.86 m/s	1
		Self-selected speed uphill (Lathouwers et al., 2022)	0.58–1.17 m/s	1
	Camber ramp	Length (Bai et al., 2017)	6 m	1
		Inclination (Bai et al., 2017)	2.5°	1
		Self-selected speed (Bai et al., 2017)	.9–1.4 m/s	1
Quiet Standing on slopes	Instrumented ramp	Inclination (Ernst et al., 2017; McGrath et al., 2018)	10° downhill to 10°uphill	2
		Duration of each trial (Ernst et al., 2017; McGrath et al., 2018)	14–15 s	2
		Trials (Ernst et al., 2017; McGrath et al., 2018)	3	2
Parcourse	Lab setup	Circuit (5 turns, 2 stairs, 1 obstacle, 1 ramp) (Barnett et al., 2021)	31.5 m	1
	Oval circuit	Indoor–outdoor walking (variations of camber walking and surface) (Graham et al., 2007; Ernst et al., 2017; McGrath et al., 2018)	207.3 m	1
Performance-based clinical outcome measures	Clinical test	2-minute walking test (Barnett et al., 2021)	n-a	1
		Time up and go test (Barnett et al., 2021)	n-a	1
		L-test (Barnett et al., 2021)	n-a	1

**Table 4.** Summary of main findings

N.	Author	Feet conditions	Knee conditions	Study design	Outcome variables	Main finding
1	Agrawal et al. (2022)	Dynamic response: Low-profile Multiaxial J-shaped	Customary (no changes)	Cross-over study	Center of pressure Trajectory	No significant differences between dynamic response foot designs Some trends suggest an effect on step symmetry and medial-lateral excursion with the multiaxial foot The movement of the COP in the frontal plane is perceived as unstable
2	Alexander et al. (2018)	Hydraulic ankle Solid ankle	Microprocessor (no changes)	Cross-over study	Spatiotemporal parameters Ground reaction force Joint kinematics Joint moments	At ramp walking while using a hydraulic ankle-foot vs a rigid ankle: Substantial reduction in hip flexion moment during the prosthetic stance phase Longer single support time of the intact side due to an increased toe clearance during the prosthetic swing phase Reduction in flexion moments could possibly lead to a reduction in energy cost
3	Alexander et al. (2020)	Hydraulic ankle Solid ankle	Participant 1: C-leg Participant 2: Orion	Case series	Spatiotemporal parameters Ground reaction force Joint kinematics	Reduction in hip moment while walking downhill when using a hydraulic ankle-foot vs a rigid ankle
4	Armannsdottir et al. (2018)	Powered feet: Before training, inactive dorsiflexion Before training, active dorsiflexion After training, inactive dorsiflexion After training, active dorsiflexion	Microprocessor customary (no changes)	Case series	Spatiotemporal parameters Pain rating Joint kinematics	By using a microprocessor-controlled feed with a training period of 6 weeks: Decreased in compensatory circumduction Decreased pelvic lift on the prosthetic side Ultimately resulted in improved symmetry
5	Bai et al. (2017)	Hydraulic ankle Solid ankle	Customary (no changes)	Cross-over study	Spatiotemporal parameters Joint kinematics Joint moments Symmetry Comfort score	The use of a hydraulic foot showed: An improved temporal symmetry index symmetry An increase in ankle range of motion A prosthetic ankle moment similar to the sound side Authors acknowledge the influence of different prosthetic knees on TSI values
6	Bai et al. (2018)	Hydraulic ankle Solid ankle Microprocessor	Customary (no changes)	Cross-over study	Spatiotemporal parameters Joint kinematics Joint moments Symmetry Questionnaire results	The hydraulic ankle-foot devices (with or without micro-processor control) showed: A relatively better bio-mimicking of the ankle moment A slightly increased range of motion Enhanced walking safety (preventing knee collapse) Facilitated a greater knee flexor moment during the whole mid-stance phase during downhill The microprocessor control: Enhances the benefits of the hydraulic ankle Better suited for users that require frequent adjustment of their prosthetic ankle properties for different surfaces
7	Barnett et al. (2021)	Hydraulic ankle Solid ankle	1) Microprocessor 2) Non-microprocessor	Cross-over study	Duration of test Walked distance	Despite the lack of statistical significance, results reflect a general benefit to mobility of using a combination of more complex prosthetic components Prosthetic knee functionality was seemingly more influential than prosthetic ankle-foot function The provision of an advanced ankle-foot component with both microprocessor and non-microprocessor knees had positive influences on mobility
8	Blumentritt et al. (1998)	Elastic keel Multiaxial elastic keel Multiaxial dynamic response Single-axis Multiaxial Dynamic response	Rotary hydraulic (no changes)	Cross-over study	Joint kinematics Joint moments	Prosthetic ankle-foot devices significantly influence subjective walking comfort for the amputee as well as the external moments about the prosthetic knee A preference for the highest energy return option if there is no need for compensation at the sound side limb Each amputee had clear and differing preferences for specific foot devices Each amputee chooses a particular prosthetic foot to complement their individual ability to generate a hip extension moment
9	Boonstra et al. (1993)	Multiaxial Dynamic response	Polycentric or hydraulic (no changes)	Cross-over study	Spatiotemporal parameters Joint kinematics	Preference seemed not to be related to physical characteristics of the amputees nor to gait parameters The multiaxial foot: Increase the adduction–abduction movement of the hip
10	De Asha et al. (2014)	Solid ankle (customary) Hydraulic ankle	Customary (no changes)	Cross-over study	Center of pressure trajectory Spatiotemporal parameters Center of mass velocity Ground reaction force Symmetry	Improvements in passive prosthetic-foot “function” may be less dependent on energy return during late stance than on reducing resistance to COP progression (“braking effect”) during early-to-mid stance An improvement in gait function and an increase in symmetry are independent from each other While using the hydraulic ankle joint: A significantly smoother/more rapid progression of the CoP – less COP forwards velocity reduction (“braking effect”) during prosthetic limb stance
11	De Pauw et al. (2018)	Solid ankle (customary) Experimental (microprocessor)	Single-axis or rotary hydraulic (no changes)	Cross-over study	Spatiotemporal parameters Foot evaluation Metabolic variables	While using the experimental (microprocessor): Increased heart rate and rate of perceived exertion at normal walking speeds, probably due to the limited acclimation period Increased intensity of effort compared to the currently existing passive prostheses
12	De Pauw et al. (2020)	Solid ankle (customary) Experimental (microprocessor)	Single-axis or rotary hydraulic (no changes)	Cross-over study	Spatiotemporal data Ground reaction force Joint kinematics	The combination of the knee and ankle joints makes complex and ambiguous biomechanical outcomes While using the experimental (microprocessor): No benefit because TFPUs do not lift the knee to a sufficient height in order to enable the unlocking mechanism A combination of both distal and proximal compensations due to the absence of ankle and knee joints
13	Ernst et al. (2017)	Microprocessor 1 Microprocessor 2 Microprocessor 3 Microprocessor 4 Non-microprocessor (customary)	Customary microprocessor: 1) Knee stance function enabled 2) Knee stance function disabled	Cross-over study	Ground reaction force Joint kinematics Joint moments	A prosthetic knee joint with lock mechanism in standing did not exhibit the weight shift to the sound side, independent of the foot used Minor adaptations in GRF distribution with an enabled knee control, but feet-dependent adaptations were observed in the knee control disabled For knee joints without stance function, a fully adapting foot concept with a dorsiflexion stop appears to be beneficial for standing on slopes
14	Ernst et al. (2022)	Dynamic response Microprocessor	Microprocessor (no changes)	Cross-over study	Ground reaction force Joint kinematics Joint moments	A coupling and coordinated control of both the microprocessor foot and microprocessor knee may further improve gait on slopes and uneven ground For descending slopes: Prosthetic knees with a yielding mechanism contributed more to safety and walking than a microprocessor foot Microprocessor foot acted like a “regular” prosthetic foot
15	Goh et al. (1984)	Single-axis Solid ankle	Single-axis (no changes)	Cross-over study	Spatiotemporal data Ground reaction force	Both types of prosthetic feet can mimic whole body kinetic patterns with the proper selection of heel stiffness and alignment of the prothesis The single-axis foot showed similar characteristics to a normal foot
16	Goujon et al. (2006)	Customary Dynamic response	Customary (no changes)	Case–control study	Spatiotemporal data Ground reaction force Joint kinematics	While analyzing the foot as two articulated rigid bodies (forefoot and hindfoot): Stiffer joint at the mid-foot allows a prolongation of prosthetic step, preventing collapse Stiffer prosthetic forefoot prevents a too high anteroposterior first peak of GRF on the sound side and allows a better control of weight transmission in the late stance phase
17	Graham et al. (2007)	Multiaxial Dynamic response	Stance flex and pneumatic swing or microprocessor (no changes)	Cross-over study	Spatiotemporal data Ground reaction force Joint kinematics Joint powers Comfort score Walked distance Duration of test	While using a dynamic response foot: A more symmetric gait pattern presumably due to greater flexibility Increase in self-selected walking speed Increase in prosthetic ankle plantarflexion power push-off which caused greatest prosthetic knee flexion at mid-swing A closer hip “normal” motion with a greater total lower-limb power output from the prosthetic side
18	Graham et al. (2008)	Multiaxial Dynamic response	Stance flex and pneumatic swing or microprocessor (no changes)	Cross-over study	Metabolic variables	The clinical significance of the results is still open to debate because the participant was highly active While using a dynamic response foot: Reduce oxygen consumption Increase in self-selected walking speed probably due to a reduced oxygen consumption
19	Hong et al. (2022)	Experimental: Flat-toe foot (FT) Curved toe (CT)	Powered	Case series	Spatiotemporal data Ground reaction force Joint kinematics Joint moments Symmetry	CT foot tended to have an earlier heel-off and a later toe-off Curvature of the CT foot allowed the subject to have a more “natural” roll-over while walking, leading to a faster load transfer during the stance phase FT foot provided greater push-off power to the user than the CT foot
20	Ingraham et al. (2014)	Foot and knee: same experimental powered prosthesis with different control strategies: Swing initiation Swing initiation + increasing ankle stiffness Swing initiation + powered plantarflexion Swing initiation + increasing ankle stiffness + powered plantarflexion Baseline		Cross-over study	Spatiotemporal data Joint kinematics Joint moments Joint powers	Powered plantar flexion: Higher positive power at the ankle in late stance, with peak values similar to able-bodied gait Higher contributions of the ankle joint to the forward propulsion and support of the body Increasing ankle stiffness: Lower stance phase dorsiflexion torque acting from initial contact to mid-stance Subjects perceived a smoother progression from heel strike to foot flat
21	Ingraham et al. (2016)	Foot and knee: same experimental powered prosthesis with different control strategies: Swing initiation Swing initiation + increasing ankle stiffness Swing initiation + powered plantarflexion Swing initiation + increasing ankle stiffness + powered plantarflexion Baseline		Cross-over study	Spatiotemporal data Ground reaction force Joint kinematics Joint moments Joint powers	Providing knee swing initiation + increasing ankle stiffness + powered plantarflexion influences the walking mechanics Gradual increase in ankle stiffness showed: Increased braking GRFs of the amputated leg because the foot achieved “foot-flat” faster due to lower initial ankle stiffness Influenced the shape of the prosthetic ankle plantarflexion moment, mirroring the intact ankle moment Increased prosthetic ankle power generation (despite a similar maximum stiffness value across conditions)
22	James and Stein (1986)	Single-axis Experimental (independent movement of heel, mid-foot, and forefoot)	N/A	Cross-over study	Spatiotemporal data Joint kinematics	The amount of dorsiflexion determines the hip trajectory following mid-stance and the timing of knee flexion moment While using the experimental joint: The amount of flexion at experimental joint affects the timing of the knee flexion Made walking up inclines easier Affected knee stability while walking on declines Made foot placement on stairs and ladders more critical due to potential knee instability
23	Klenow et al. (2016)	Dynamic response: Continuous lever on carbon fiber Single spring on carbon fiber Integrated spring on glass composite Continuous lever on glass composite Hydraulic ankle on carbon fiber	N/A	Cross-over study	Center of pressure trajectory Dead spot phenomenon metrics Spatiotemporal parameters	The CoP “dwell” in the hindfoot contributed to resistance of the prosthetic shank to rotate forward which prolonged the posteriorly directly portion of the anteroposterior GRF and its magnitude A marked reduction in CoP velocity, and not solely posterior CoP displacement, may constitute the presence of a death spot phenomenon during gait with a particular prosthetic foot The effect of different designs on death spot phenomenon is still not clear
24	Lathouwers et al. (2022)	Participants Experimental (passive, Talaris demonstrator)	Customary (no changes)	Cross-over study	Spatiotemporal data Metabolic variables Duration of test Comfort score	The Talaris demonstrator increased comfort and decreased fatigue attributed to the flexibility of stiffness of the experimental articulated ankle joint
25	Lathouwers et al. (2023)	Participants Experimental (passive, Talaris demonstrator)	Customary (no changes)	Cross-over study	Join kinematics Spatiotemporal data	Individual comparisons of TFPUs suggest an advantage of the Talaris demonstrator by reducing the standard deviation between steps of hip-knee and knee-ankle angles mimicking the behavior of able-bodied individuals
26	Lee and Hong (2009)	Experimental: Solid ankle Single-axis	Experimental microprocessor (no changes)	Cross-over study	Joint kinematics Joint moments	The knee moment resembles the behavior of “natural” knees with a solid ankle Smaller impact energy absorption by single-axis ankle The mobility of the foot-ankle system controlled the relative knee flexion–extension angle of the knee which impacted the energy absorption of the knee
27	Macfarlane et al. (1997)	Dynamic response Solid ankle	Stance and swing with hydraulic control (no changes)	Cross-over study	Spatiotemporal data Symmetry	While using the dynamic response foot: Took longer to clear the prosthetic foot off the ground, delaying the swing phase Reaction force at late stance (push-off) facilitates lifting the lower segment of the prosthesis More symmetrical late stance phase
28	Macfarlane et al. (1997)	Dynamic response Solid ankle	Stance and swing with hydraulic control (no changes)	Cross-over study	Metabolic variables	While using the dynamic response foot: Lower exercise intensity Lower energy costs More efficient gait across a functional range of speeds
29	McGrath et al. (2018)	Hydraulic ankle Solid ankle	Microprocessor: 1) Standing support activated 2) Standing support deactivated	Cross-over study	Center of pressure trajectory Ground reaction force Joint kinematics Symmetry	The ankle mechanism enables the joint to have a variable equilibrium position and thus “self-align” within the range of hydraulic movement, permitting a more natural posture with reduced kinematic compensations Microprocessor-controlled standing support mode at the knee and hydraulic self-alignment of the ankle joint contributed to more symmetrical weight distribution and improved balance ability Both prosthetic knee and ankle technologies must be considered when assessing the optimum clinical outcomes for TFPUs
30	McNealy and Gard (2008)	Multiaxial Solid ankle	Customary (no changes)	Cross-over study	Spatiotemporal data Joint kinematics Joint moments Joint powers Foot evaluation Comfort score	No differences in experimental data between conditions Participants perceived increased comfort with the multiaxial foot because of increased motion during the stance phase The multiaxial foot increased range of motion but did not alter the temporal–spatial parameters
31	Pace et al. (2020)	N/A	N/A	Numerical simulation	Ground reaction force Joint kinematics Joint moments	Identified solution spaces for alignment and ankle-foot stiffness during the swing phase Posterior to in-line knee alignment with low to medium ankle-foot stiffness improves knee stability in early and mid-stance whereas facilitating knee break at the end of the stance phase Anterior knee alignment and rigid feet are a threat to the stability of TFPUs
32	Pröbsting et al. (2022)	Solid ankle Dynamic response Powered: 3.1) No power 3.2) Low power 3.3) Optimal power	Microprocessor (no changes)	Cross-over study	Spatiotemporal data Ground reaction force Joint kinematics Joint moments Joint powers	Optimal ankle power showed a reduction in the knee loading parameters at the sound side especially at the knee flexion moment Similar behavior between the passive feet with the power device at no power Importance of foot construction on ankle power devices for the load on the sound side knee
33	Sedki and Moore (2013)	Multiaxial Dynamic response	Customary (no changes)	Case report	Foot evaluation	For the hydraulic response foot, questionnaire results reported: Marginal improvement of “transferring,” “utility,” and “well-being” Significant improvement in relation to walking (the “ambulation” and “gait” domains) and to the patient’s overall impression of the prosthesis
34	Sensinger et al. (2013)	Experiment: Dynamic foot simulation: Single-axis knee with 0° ankle dorsiflexion Single-axis knee with 5° constant ankle dorsiflexion Single-axis knee with ankle dorsiflexion that increased with knee flexion	Experiment and simulation: 1) Single-axis 2) Polycentric	Cross-over numerical simulation	Joint kinematics Toe clearance	Prosthetic components, such as ankle mechanisms that provide dorsiflexion during mid-swing (5° or greater), improve toe clearance to a greater extent and should be used either as replacements for or in conjunction with four-bar mechanisms for attaining maximum toe clearance
35	van der Linden et al. (1999)	Dynamic response 1 Dynamic response 2 Dynamic response 3 Multiaxial	Single-axis	Case series	Spatiotemporal data Ground reaction force Joint kinematics Joint moments Joint powers	The construction of a dynamic response foot impact on one or more kinematic and kinetic parameters A greater first peak of vertical GRF on the sound side while using a dynamic foot with a smaller dorsiflexion angle which impacted the vertical motion of the body

## 4. Discussion

The aim of this review was to summarize the current knowledge about the effects of prosthetic ankle-foot mechanism design on gait and standing performance in TFPUs. The 35 articles included in this review covered numerical simulation studies and human performance studies that tested the effects of properties from both commercial and experimental ankle-foot mechanisms. To best summarize effects on performance, this discussion has been divided into sections according to the task for which performance was assessed (Figure 5).

### 4.1. Straight-line level walking

The majority of investigations included in this review (77%) included assessment for straight-line walking over level ground. Seven studies directly adjusted the loaded prosthesis behavior through: the addition of an articulation at the metatarsophalangeal joint (James and Stein, 1986) (connecting the forefoot and midfoot through a laminated leaf spring), modifying the sagittal (unloaded) profile of the keel (Hong et al., 2022) (flat-toe shape versus curved-toe shape), or the addition of an ankle articulation (Goh et al., 1984; Lathouwers et al., 2022; Elke Lathouwers et al., 2023; Lee and Hong, 2009; McNealy and Gard, 2008). Although different approaches, each technique ultimately affects the prosthetic ankle-foot ROS radius, which effectively provides a spatial mapping of how the leg is guided through stance (A. H. Hansen and Childress, 2010). The studies that facilitated the motion of the terminal rocker through either a metatarsophalangeal joint or curved toe generally reported improved gait dynamics: a metatarsophalangeal joint promoted forward progression of the plantar center of pressure (COP), hip motion symmetry, and hip control at mid-stance (James and Stein, 1986), while a curved-toe shape delivered a smoother COP progression in early stance and longer load transfer to the contralateral limb (increased first double support) (Hong et al., 2022). The addition of an ankle articulation increased the ankle dorsiflexion–plantarflexion range of motion (McNealy and Gard, 2008) and provided a smoother ROS progression (Goh et al., 1984), which was considered better at mimicking the behavior of an anatomical ankle-foot complex. These results in TFPUs align with studies on TTPUs (A. H. Hansen et al., 2006; M. J. Major et al., 2014; Vaca et al., 2022), which observed improvement in step length symmetry and reduction in the first peak of the GRF on the prosthetic limb with a closer-to-biological ROS radius. However, an ankle articulation in TFPUs also reduced prosthetic ankle power peaks (McNealy and Gard, 2008) compared to non-articulation and produced an external knee flexion moment while using a stance phase-controlled knee prosthesis (Lee and Hong, 2009). Overall, an appropriate ROS radius may aid forward progression of TFPUs, but its effect on knee flexor-extensor moments requires consideration beyond that relevant for TTPUs.

Several studies compared commercial DR ankle-feet to non-DR ankle-feet (i.e., SACH, Multiflex) (Boonstra et al., 1993; Macfarlane et al., 1997; Macfarlane et al., 1997; Goujon et al., 2006; Graham et al., 2007; Graham et al., 2008; Pröbsting et al., 2022) and/or across different commercial DR ankle-feet designs (Blumentritt et al., 1998; van der Linden et al., 1999; Klenow et al., 2016; Agrawal et al., 2022) (i.e., varied by geometry, construction, or material). The mechanical benefits of the DR feet were similar between TTPUs and TFPUs (Goujon et al., 2006; Agrawal et al., 2022), which include better energy management during gait and aiding the body’s transition into the swing phase. Compared to non-DR feet, a DR foot was observed to increase walking speed (Macfarlane et al., 1997; Macfarlane et al., 1997; Goujon et al., 2006; Graham et al., 2007; Graham et al., 2008), improve temporal–spatial symmetry (Boonstra et al., 1993; Goujon et al., 2006; Graham et al., 2007) (step length, stance time, and hip joint kinematics), increase prosthetic side hip power generation at pre-swing (Graham et al., 2007), and reduce metabolic energy consumption (Macfarlane et al., 1997; Graham et al., 2008). Across DR ankle-feet, there were observed differences between designs, such as slower forward COP progression velocity associated with an integrated spring design that would hinder limb advancement during stance (Klenow et al., 2016); greater medial-lateral COP excursion during the mid-to-late stance associated with a split keel design to aid controlled body weight transfer to the contralateral limb during double support (Agrawal et al., 2022); and a larger sound side first GRF peak associated with the Seattle LightFoot (van der Linden et al., 1999). In early stance, a carbon fiber DR foot reduced sound side knee compensations when compared with other DR and non-DR designs (Blumentritt et al., 1998), suggestive of TFPUs’ ability to generate a hip extensor moment to modulate the prosthetic knee external moment and stabilize the prosthetic leg. Overall, these articles identified several advantages of using DR feet over non-DR feet and how individual DR designs may affect certain gait features.

Uniquely, two studies compared solid DR ankle-feet with and without a hydraulic ankle unit (De Asha et al., 2014; Bai et al., 2017). The use of a hydraulic ankle was associated with a smoother progression of the plantar COP during stance, resulting in less body center-of-mass velocity reduction (De Asha et al., 2014). Additionally, similar to the results of adding an ankle articulation (Goh et al., 1984; McNealy and Gard, 2008; Lee and Hong, 2009), and mirroring results in TTPUs (De Asha et al., 2013; De Asha et al., 2014), the inclusion of a hydraulic ankle increased ankle-foot range of motion, self-selected walking speed, and bilateral symmetry in the ankle dorsiflexion–plantarflexion moment (Bai et al., 2017).

Besides studies comparing across purely passive-elastic components, four studies (Armannsdottir et al., 2018; De Pauw et al., 2018; De Pauw et al., 2020; Pröbsting et al., 2022) compared walking performance between passive-elastic ankle-foot prostheses and microprocessor ankle-foot prostheses that deliver active plantarflexion (i.e., late stance power generation) (Pröbsting et al., 2022), active dorsiflexion (Armannsdottir et al., 2018), and variable ankle impedance across the stance phase to generate dorsiflexion–plantarflexion moments that resembled biological ankle dynamics (De Pauw et al., 2018; De Pauw et al., 2020). Delivery of active late-stance plantarflexion was associated with smaller sound side vertical GRF, external knee adduction moment, and external flexor moment compared to a SACH foot, but only a smaller external flexor moment compared to a DR foot (Pröbsting et al., 2022). Active dorsiflexion during the swing phase increased bilateral hip joint ROM symmetry and reduced prosthetic side hip circumduction, likely due to the increased ground clearance afforded by a dorsiflexed foot (Armannsdottir et al., 2018). Variable ankle impedance was found to increase perceived exertion compared to using a SACH foot during walking at a self-selected speed but revealed no other differences in gait kinematics and temporal–spatial parameters (De Pauw et al., 2018; De Pauw et al., 2020), which authors attributed to the unique walking mechanics of TFPUs that interrupted the active plantarflexion release mechanism of the prosthesis. Accordingly, the mixed results comparing DR to microprocessor-controlled ankle-feet may be related to the gait patterns specific to TFPUs involving the intact hip and prosthetic knee joints that manage ground loading during the transition phases (i.e., early stance phase and pre-swing), which are used as inputs for control schemes.

Finally, three studies (K. A. Ingraham et al., 2014; K. M. Ingraham et al., 2016; Pace et al., 2020) also included prosthetic knee joint adjustments in combination with different ankle-foot mechanisms on TFPU walking performance: comparing in-vivo different control strategies (i.e., swing initiation, increasing ankle stiffness, powered plantarflexion, and no active control) of an experimental combined knee-foot prosthesis (K. A. Ingraham et al., 2014; K. M. Ingraham et al., 2016) and analyzing in silico the effect of single-axis prosthetic knee joint center of rotation position and prosthetic ankle-foot stiffness (modeled as ROS curvature) (Pace et al., 2020). While using the experimental knee-foot prosthesis, active plantarflexion increased ankle power generation, similar to the effect of commercial powered feet on TFPUs (Pröbsting et al., 2022) and TTPUs (Mazzarini et al., 2023), while reducing prosthetic side braking via smaller average posterior GRF (K. M. Ingraham et al., 2016). Variable ankle impedance to mimic biological stance behavior was shown to increase walking speed (K. A. Ingraham et al., 2014), While aiding swing initiation through active plantarflexion and increased ankle stiffness not only increased gait speed but also reduced ipsilateral hip joint compensations (K. A. Ingraham et al., 2014; K. M. Ingraham et al., 2016). Similarly, the numerical simulation demonstrated that knee flexion–extension moments could be manipulated through combined adjustments of prosthetic knee alignment and ankle-foot mechanics to optimize the total leg design for limb stability during mid-stance and transition from late stance into swing (Pace et al., 2020). These results illustrate the important interaction effects of prosthetic ankle-foot and knee mechanisms on TFPU gait (K. A. Ingraham et al., 2014; K. M. Ingraham et al., 2016; Lee and Hong, 2009), which warrants further research to better inform prescription guidelines.

### 4.2. Straight-line gradient walking

Six studies investigated the effects of different prosthetic foot designs on gradient walking in TFPUs. One study modified elements of the ankle-foot mechanism by adding an articulation at the metatarsophalangeal joint of a single-axis foot and studied gait with and without the additional joint (James and Stein, 1986). The metatarsophalangeal joint modified the dorsiflexion–plantarflexion motion to better resemble able-bodied dynamics and reduced an excessive second elevation of the hip to yield increased bilateral hip motion symmetry. The increased motion allowed the prosthetic foot to achieve full plantar contact with the surface with less generation of external plantarflexion moments but created an earlier knee flexion due to the anterior progression of the COP due to the increased dorsiflexion, which was perceived as unstable by the participants.

Studies compared the use of a hydraulic ankle DR foot to a solid ankle DR foot, reporting improvement in step length symmetry (Bai et al., 2017, 2018), differences in ankle moment peaks (Bai et al., 2018), and reduction in the hip extension moment (Alexander et al., 2018) during incline and decline walking, with a reduction in hip ROM (Alexander et al., 2018; Alexander et al., 2020) during decline walking. The improvement in step length symmetry for TFPUs was associated with increased toe clearance due to the retained dorsiflexion of the hydraulic ankle during the swing phase, which has also been reported for TTPUs (Riveras et al., 2020). Moreover, a hydraulic ankle DR foot achieved full plantar surface contact to the sloped surface, reducing the effect of ankle external moments due to the bending of the DR foot. The reduction in ankle external moments attributed to the GRF vector direction resulted in a smaller lever arm at the hip joint, so a smaller hip extension moment was needed to maintain limb stability. This effect does not appear to be limited only to sagittal plane slopes, since an improvement in step length symmetry was also identified when TFPUs walked on a coronal plane slope (i.e., camber walking) (Bai et al., 2017). Two manuscripts (Bai et al., 2018; Ernst et al., 2022) investigated hydraulic ankles by examining the effect of a microprocessor that controlled stance-phase articulation impedance. Compared to a solid ankle DR foot during incline walking, a microprocessor-controlled ankle-foot resulted in a reduction in the prosthetic knee extension moment (by 26% at vertical shank orientation) (Ernst et al., 2022), less hip joint ROM, and improved ankle moment symmetry (Bai et al., 2018). In summary, the hydraulic ankle-foot independent of the microprocessor control increased toe clearance and improved symmetry on different slopes compared to solid ankle DR feet, and the microprocessor control could further enhance the benefits of the hydraulic ankle (Bai et al., 2018) through more controlled management of ankle dorsiflexion–plantarflexion angle adaptation to different slopes.

Finally, as with level walking, an interaction between the prosthetic knee and ankle-foot components was reported across the gradient walking studies: an increase in knee flexor moment due to a solid ankle (Ernst et al., 2022), an increase in ankle-foot ROM due to the timing of the prosthetic knee flexion initiation (James and Stein, 1986), and the modification of the GRF vector direction and COP progression by a knee with a gradual yielding mechanism (Alexander et al., 2020). Again, further research is warranted on the interaction effects of different prosthetic knee and ankle-foot mechanisms during gradient walking to inform prescription guidelines for accommodating community ambulators that may traverse terrain beyond level ground walking.

### 4.3. Quiet standing on slopes

Two studies evaluated quiet standing on slopes by comparing DR feet with and without a hydraulic ankle joint (McGrath et al., 2018) and four commercially available microprocessor-controlled ankle-feet to a passive-elastic ankle-foot mechanism (Ernst et al., 2017). The first study described how a hydraulic ankle with variable damping enables the ankle joint to adapt to a neutral position during slope standing, resulting in a reduction in compensatory strategies at the hip on the prosthesis side in TFPUs. Meanwhile, the second study determined that the ability of microprocessor feet to lock once fully adapted to the inclination and the increase ankle ROM improved bilateral weight distribution of TFPUs while standing on a decline slope. Both studies included evaluations while using a microprocessor-controlled knee with standing support assistance activated and deactivated. With standing support activated, differences in weight shifting and COP motion between hydraulic, microprocessor-controlled, and passive ankle-foot mechanisms were mitigated, suggesting that the knee mechanisms could affect hip compensations. This evidence suggests that ankle-foot mechanisms capable of adapting to different slopes could achieve better functionality during standing, but the combined effect of both prosthetic knees and ankle-feet should be considered as recommended with walking.

### 4.4. Parcourse

Evaluations of prosthesis users in scenarios reflecting the lived environment are often desirable to determine how prosthetic components function outside of controlled clinical or research laboratory settings. Two studies (Graham et al., 2007; Barnett et al., 2021) assessed TFPU mobility performance when traversing a parcourse that included level ground, slopes, stairs, obstacles, and turning. The results reported that a hydraulic ankle DR foot performed better than a solid ankle DR foot (Barnett et al., 2021), and there were no observed differences between a solid ankle DR foot and a multiaxial foot (Graham et al., 2007) when comparing completion times of a parcourse circuit. The hydraulic component aided TFPUs’ gait through adaptation to the different terrains, which again supported the other studies in this review that a hydraulic ankle DR foot may be beneficial to overall TFPUs’ ambulation more generally.

### 4.5. Performance-based clinical measures

Only one study (Barnett et al., 2021) compared user performance between a hydraulic ankle DR foot and a solid ankle DR foot in combination with a microprocessor-controlled and non-microprocessor-controlled prosthetic knee using established performance-based clinical outcome measures (two-minute walk test, timed up and go, and L-test). The hydraulic ankle DR foot improved performance across all clinical measures independent of the prosthetic knee, suggesting generally improved mobility capability of TFPUs. Additionally, this result aligns with the previously discussed gait studies where a hydraulic ankle DR foot could have meaningful benefits to TFPU mobility and complement the functionality of an appropriate prosthetic knee design.

## 5. Limitations

There are limitations to this literature review that should be considered when interpreting its results. Only one author extracted, classified, and summarized the information presented in this review, but resolving issues about the eligibility of articles and analysis of their results were performed in collaboration with three authors. Additionally, the authors did not perform a critical appraisal of research quality involving the selected articles. This review also inherits the limitations from the selected articles. All the human subject testing included in this review included ten participants or fewer. Therefore, as a result, the studies may have been underpowered, and the participant cohort may have limited generalizability. Finally, several studies were excluded because the grouped results included both TTPUs and TFPUs and precluded evaluation based on amputation level.

## 6. Conclusions

This review aimed to summarize the state of the literature describing the effects of prosthetic ankle-foot design on TFPU walking and standing performance and to serve as a complement to existing knowledge on TTPU effects. The reviewed literature utilized a variety of protocols, device comparisons, and outcome variables, rendering it challenging to summarize results. However, the results generally suggest many parallels with TTPUs, such as improvements in walking performance related to the incorporation of biological ROS features, energy storage and return capability, and especially a hydraulic ankle (active or passive) for adapting to non-level terrain, which also demonstrated benefits during quiet standing on slopes. The literature also emphasized the need to consider the interaction between prosthetic ankle-foot mechanics and the prosthetic knee, particularly how the ankle-foot component directs the GRF vector and plantar COP to affect knee moments, which stands apart from TTPUs who have the advantage of volitional management of intact knee joint moments. The interaction effects of prosthetic ankle-foot and knee mechanisms require additional research to develop a fuller understanding of how to best match an ankle-foot and knee design, so the two components synergistically operate to offer the best performance of both, thereby informing this critical aspect of TFPU clinical practice guidelines.

## Supporting information

10.1017/wtc.2025.10037.sm001 Vaca et al. supplementary material Vaca et al. supplementary material

## Data Availability

Data can be made available to interested researchers upon request by email to the corresponding author.

## Supplementary material

The supplementary material for this article can be found at http://doi.org/10.1017/wtc.2025.10037.
